# The Impact of Human Activities on Zoonotic Infection Transmissions

**DOI:** 10.3390/ani13101646

**Published:** 2023-05-15

**Authors:** Michelle Marie Esposito, Sara Turku, Leora Lehrfield, Ayat Shoman

**Affiliations:** 1 Department of Biology, College of Staten Island, City University of New York, Staten Island, New York, NY 10314, USA; 2 Ph.D. Program in Biology, The Graduate Center, City University of New York, New York, NY 10314, USA; 3 Macaulay Honors College, City University of New York, New York, NY 10314, USA

**Keywords:** zoonoses, emerging, climate change, urbanization, deforestation, wildlife exploitation

## Abstract

**Simple Summary:**

Human activities, such as changing the environmental landscape or developing animal-related tourist attractions and zoos, have greatly increased the interactions between humans and animals. Due to this increase in direct contact between humans and animals, the risk of transmission of infections called zoonoses, meaning originating in animals, has increased. We aim to review how human activities have driven zoonoses and why it is so important to study this topic in order to help develop preventative measures against further zoonotic infections in the public.

**Abstract:**

As humans expand their territories across more and more regions of the planet, activities such as deforestation, urbanization, tourism, wildlife exploitation, and climate change can have drastic consequences for animal movements and animal–human interactions. These events, especially climate change, can also affect the arthropod vectors that are associated with the animals in these scenarios. As the COVID-19 pandemic and other various significant outbreaks throughout the centuries have demonstrated, when animal patterns and human interactions change, so does the exposure of humans to zoonotic pathogens potentially carried by wildlife. With approximately 60% of emerging human pathogens and around 75% of all emerging infectious diseases being categorized as zoonotic, it is of great importance to examine the impact of human activities on the prevalence and transmission of these infectious agents. A better understanding of the impact of human-related factors on zoonotic disease transmission and prevalence can help drive the preventative measures and containment policies necessary to improve public health.

## 1. Introduction

Human activities, such as urbanization, deforestation, wildlife exploitation, and tourism, as well as the global climate changes that have occurred from mankind’s inhabitation of the planet, not only change the landscapes of nature but also serve as driving forces of zoonotic disease emergences, thereby increasing the prevalence of already known zoonoses [1]. Various animals and arthropod reservoirs have been linked to the connection between human-related factors and the emergence of diseases, including rodents, birds, pigs, cows, bats, primates, camels, mosquitoes, ticks, and fleas [1,2]. It is important to study the various causes of emerging zoonoses, as these diseases account for more than 60% of infectious diseases encountered by humans and can create worldwide devastation as seen during the COVID-19 pandemic [3,4]. As mankind becomes more aware of the threats of pandemics, such as the plague, Spanish flu, and SARS-CoV-2, it is of great interest to better characterize ways to minimize activities that increase reservoirs or human–animal contact [3]. While it is seemingly impossible to completely stop new infections from spreading from animals to humans, it may be possible to reduce the severity of risks to the human population via quicker or more efficient methods of detection, early warning systems, and proper control or prevention policies if we better understand the activities that influence or drive these zoonotic transmissions [5]. While other reviews tend to focus on anthropogenic factors of land use changes influencing zoonoses or wildlife exploitations and impacts in separate reviews, we aim to make a more comprehensive exploration of these factors together as one big-picture view. By shedding light on human-related factors that increase zoonotic risks in this review, we aim to demonstrate the factors to consider when developing prevention and containment plans, such as urban risk management, pest policies, sanitation protocols, and public health awareness campaigns.

## 2. Urbanization

With urbanization comes many changes, such as demographic growth, migration of people and animals, and changes in land usage, that promote the potential for zoonoses [6]. Loss and modification of habits can drive animals to develop new patterns of human interactions [7]. Multidisciplinary, ecosystem-level analyses of Kuching, Malaysia, have demonstrated that urbanization creates a cascading effect of modifications to animal reservoirs, including arthropod vectors and microbes, which increases emergence risks, especially with regard to environmental and tick-borne infection transmissions [8]. Two species of rodents associated with pathogen transmission and observed in high concentrations in urbanized regions are *Rattus rattus* and *Sundamys muelleri* [8]. *Rattus rattus* contributes to environmental zoonoses, such as *Leptospira* spp., in which *S. muelleri* contributes to tick-borne zoonoses via *Amblyomma*, *Haemaphysalis*, and *Ixodes* [8]. Rodents are amongst the most studied reservoirs in relation to urbanization. One very large study of a 48-year-long dataset of rodent-borne hemorrhagic fever-inducing hantaviruses in China, for instance, performed spatiotemporal analyses of these hemorrhagic fever incidences in relation to urbanization, economic growth rates, geographic expansions, and migrations of human populations [9]. This study demonstrates a high correlation between the number of urban immigrants and human incidence of zoonotic disease, but also reveals an inverted U-shaped relationship between incidence and urbanization as an endemic turning point from interventions [9].

It should be noted that tropical and third-world countries are most susceptible to zoonotic emergence from urbanization due to their lack of infrastructure and minimal sanitation protocols [8,10]. For instance, a comparison of shack dwellers versus brick house dwellers exposed to rodents demonstrated slightly lower seropositivity for toxoplasmosis and leptospirosis, thus yielding reduced exposure to brick house dwellers in more formal sanitation conditions [10]. *Toxoplasma* and *Leptospira* are two prioritized zoonotic pathogens carried by rodents that are not only found in residential dwellings, but also frequently found on pig and dairy farms and observed in domestic cats [11]. *Leptospira* is spirochaete bacteria that causes an acute febrile illness, whereas *Toxoplasma* is a protozoan parasite responsible for chronic infection with strong implications for the overactivation of the immune system and is capable of crossing the blood–brain barrier [11,12,13]. As seen in shack dwelling versus brick dwelling results in urbanization, socioeconomic status plays a major role in the potential for zoonotic transmission [14]. Intestinal parasitic infections are known to be especially prone to socioeconomic influences, with ascariasis, trichuriasis, giardiasis, and amoebiasis being amongst the parasitic zoonoses most strongly associated with urbanization in developing countries [14]. In urban areas with rapidly developing economies, the factors that lead to zoonotic diseases end up labeled as especially acute [15]. These drivers of zoonoses include livestock trade, human distribution and density, and trade/travel networks [15]. Since socio-economic aspects of urbanization can drive zoonotic risks, these aspects should be viewed as factors to consider when developing urban risk reduction management strategies, including pest management, public health awareness, and environmental hygiene campaigns [8,10,16].

With urbanization comes a variety of connected activities that will be further discussed in this review, such as habitat destruction, pollution, climate change, and human exploitation of animals, all of which have been linked to a significant loss of biodiversity and increased risks of zoonotic exposures [7]. With the West Nile virus and Lyme disease as models, it has been demonstrated that areas with less urbanization or anthropogenic activities that retain high biodiversity subsequently have pathogen vectors feeding on a wider range of hosts that end up as poorer reservoirs for the pathogens, yielding a reduced prevalence of zoonotic emergences [7]. When biodiversity is low, however, the pathogen vectors can feed on select primary reservoirs that dominate and increase the spread of diseases [7]. Some taxa prone to zoonotic reservoirs have been found to preferentially thrive in human-dominated landscapes, whereas non-reservoirs have been found to dominate less-disturbed landscapes [17]. Ultimately, urbanization is a major driver of decreased biodiversity, as some animals have difficulty adapting to the changing environment [18]. The changed environment also contributes to some other animals being more attracted to migration into urban habitats, as these locations provide new robust food sources and protective shelters [18]. This, in turn, leads to an increase in the population of these animals as they thrive in the resource-rich urban location, which subsequently increases potential interactions with humans, and exposure of humans to the parasites or pathogens carried by these wild animals [18]. For instance, rodents, bats, Carnivora (such as dogs and cats), Cetartiodactyla (such as hooved mammals), and primates are major reservoirs of zoonotic pathogens and have all been pushed into increased human contact through urbanized landscapes or urban practices [18]. However, it should be noted that in some cases, the opposite pattern is observed in which increased biodiversity is associated with a greater prevalence of disease through a higher diversity of potential hosts to carry pathogens [19]. Urbanization, however, tends to reduce human contact with the biodiverse forest habitats in these scenarios, and thus, more studies have demonstrated the previously mentioned correlation between lower biodiversity and higher zoonotic emergences [19]. Furthermore, some studies attribute urbanization to reduced disease through increased sanitation processes, increased medical care, public health initiatives such as arthropod spraying, and improved pest reduction indoors [19].

While many pathogens and reservoirs exist, one category with significant consequences from urbanization is parasites [14,20]. In many developing countries, urbanization has included a strain that leads to insufficient water/food supplies, sanitation, garbage removal, health care, and hygiene, leading to prime conditions for parasitic contaminations or transmissions [14,21]. In one study on Sri Lankan macaques of three subspecies in urban, suburban, and wild populations, twenty gastrointestinal parasite genera were analyzed, including protozoans, cestodes, nematodes, trematodes, and acanthocephalans [20]. The results demonstrated that in all subsets of species analyzed, macaques of urban and suburban populations exhibited greater parasitic prevalence, ova and cyst counts, and species richness in comparison to samples from the wild populations [20]. Not only were the data of parasitic robustness in these macaques valuable for understanding anthropogenic impacts, but so were the observational findings of modified daily activities, ranging patterns, and food/water selection of the macaques in urban sites compared to the wild sites [20]. The forms of urbanization can also impact risk levels, as multiple helminth-infected samples of urban sites were obtained in religious or archaeological sites of the region where many pilgrims and tourists walk barefoot and are more susceptible to soil-transmitted zoonoses [20]. In addition to soil-transmission zoonoses, protozoan-infected samples were found in urban sites where macaques were seen bathing and drinking from shared water sources of humans, which increases oral route transmission risks [20]. Even burrowing mite parasites have been found to have an increased prevalence in larger urban regions rather than in wild habitats [22]. For instance, in Estonia, a higher percentage of sarcoptic mange from the parasitic mite, *S. scabiei*, was found in larger urban towns than in wild habitats [22]. It is believed that more urban locations promote the increased presence of zoonotic infected wildlife, as the infected animals require a larger amount of energy; thus, they flock towards the increased food, warmth, and shelter of urban environments [22].

## 3. Deforestation

Deforestation is another consequence of human population expansion, which in turn has been linked to an increased risk of zoonoses. As with the other factors described so far, no single factor contributes to infectious disease spread in a self-contained manner; rather, all of these human activities work together towards increasing the risk of transmission. Interestingly, while most studies focus on how deforestation leads to an increase in zoonoses, one study also pointed out that as deforestation increases the incidence of some infections, these infections can also subsequently decrease deforestation [23]. In the case of malaria, it is believed that the malaria burden subsequently reduces forest clearing through socioeconomic mechanisms, depending on the stage of deforestation [23]. In that study, it was determined that in the span of thirteen years across 795 municipalities in the Amazon basin, a 10% increase in deforestation was correlated with a 3.3% increase in malaria incidence, while a 1% increase in malaria incidence was correlated with a 1.4% decrease in cleared forest area [23].

**Figure 1:**
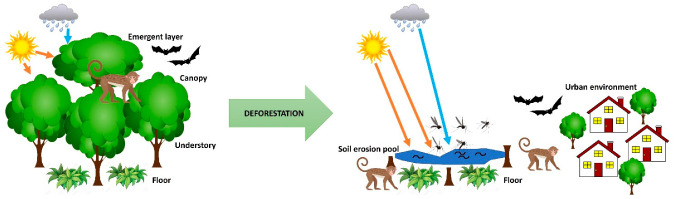
As deforestation removes the higher levels of forest structure, including the canopy, light, and precipitation, exposure to the previously protected floor level leads to soil erosion and pool formations. The new environmental landscape promotes vector reproduction and expansion. Furthermore, organisms dependent on canopy habitation are now redistributed to ground levels closer to human interactions and have increased migrations towards urban developments. Increased vectors and increased human–animal exposures contribute to an increase in infection transmissions.

Overall, however, it is more common to study the impact of deforestation on zoonoses rather than the impact of zoonoses on deforestation. Malaria-causing pathogens are amongst the microbes that have been studied in various places across the globe to determine the impact of deforestation on infection transmissions. *Plasmodium knowlesi* malaria is a vector-borne zoonosis in Southeast Asia that infects macaques and subsequently humans as well [24]. *Plasmodium knowlesi* is now known as one of the most common causes of malaria in parts of Indonesia and Malaysia, which are hotspots for deforestation [24]. Environmental changes have been discovered to affect the behavior and distribution of mosquito vector species and human populations, which helps to create new opportunities for *Plasmodium knowlesi* transmission globally [25]. The expansion of zoonotic malaria is seen with rapid deforestation across Southeast Asia, as the loss of forest canopies creates an ideal environment for the reproduction of mosquito vectors that prefer sunlit pools available after canopy removal, as well as bringing infected macaques down to the ground level near humans [25,26]. Most people, when they visualize deforestation, simply visualize the loss of trees as single entities, but tend to forget that within those trees there are various layers of ecological life dependencies, with the canopy being a very different environment from the floor level; thus, the loss of such a complex structure results in a major cascade of consequences with a completely new landscape (Figure 1). Without the canopy, the floor level and soil are no longer shielded from direct rainfall, and soil erosion is observed, as well as a modification in sunlight that is no longer obstructed [27]. This promotes the growth of aquatic vegetation and algae with which mosquitoes thrive; thus, there is an increase in mosquito-borne malaria, as well as other mosquito-carried infections [27]. Dengue fever, for instance, is another known zoonotic disease that is transmitted when a mosquito bites wildlife or humans with dengue virus in their blood [28]. Dengue fever first emerged after World War II, with rapid urbanization in Southeast Asia, which led to an increase in transmission and hyperendemicity of the virus as canopies diminished and landscapes began to promote an aquatic vegetation-rich environment favored by mosquitoes [29]. Yellow fever infection rates are also found to be associated with this trend as rates are significantly higher in areas with high levels of deforestation due to canopy-related driving factors [26,30]. Yellow fever is an acute viral disease spread by the *Aedes aegypti* mosquito [31]. Yellow fever patients go on to develop jaundice, bleeding, shock, organ failure, and death [32]. Deforestation has been credited with the resurgence of this zoonotic disease as it was originally contained in the canopy of forests labeled as an enzootic cycle between mosquitoes and monkeys; however, when trees were cut down, the contagion was transported to the forest floor where it was then passed to human hosts [26] (Figure 1).

Another way in which deforestation drives zoonoses or infectious disease transmissions is by removing native habits, and thus encouraging the movement of animals into human-inhabited regions previously unexplored by wildlife [33]. This driving mechanism has been linked to the increased spread of Ebola in some regions [34]. Ebola virus disease (EVD), first identified in Africa in 1976, causes severe inflammation, hemorrhagic fever, and tissue damage throughout the body [34]. EVD is a complex zoonosis ongoing in West Africa, with the Ebola virus found primarily in bats and primates [35]. It has been found that three different species of bats, *Hypsignathus monstrosus*, *Myonycteris torquate*, and *Epomops franqueti*, are identified as reservoirs for EVD [35]. Deforestation has been linked to an increased probability of EVD outbreaks through disruption of the natural habitats of these bat species and subsequent migrations into areas previously not explored by these animals, increasing human contact with these zoonotic viruses [33,34]. Lyme disease is also associated with this mechanism of movement. Lyme disease, first identified in Lyme, Connecticut, in 1975, is characterized by rheumatoid arthritis-like symptoms [36,37]. It was found that Lyme disease is caused by the bacterium, *Borrelia burgdorferi*, that is spread by the bite of infected black-legged ticks, frequently passing the pathogen from white-tailed deer to humans [38]. The disease is spread once a tick bites a human and stays attached for about thirty-six to forty-eight hours [39]. Deforestation has caused major changes to environmental landscapes that subsequently alter the populations and movement patterns of the principal host species, the white-tailed deer, including human exposure [40]. More recently, extensive reforestation in places such as the northeastern US has led to a major increase in deer and white-footed mouse populations and expansion into more residential locations, which has increased exposure to Lyme disease in places such as New York, New Jersey, and Pennsylvania [40,41]. Interestingly, Nipah virus (NiV), which emerged from fruit bats and can be transmitted to humans, was also greatly effected by deforestation and the novel movements of animals consequently [42]. NiV is found to cause encephalitis (swelling of the brain) and potential death [42]. The virus first emerged when *Pteropus* bats affected by deforestation began to settle in barns where they spread the virus to pigs, which in turn infected humans [43].

Deforestation modifies wildlife communities, and thus increasing zoonotic spillover potential is a growing area of concern [44]. The increase in zoonotic outbreaks, majorly in tropical lands, has been in part connected to human population growth from 1990 to 2016 [45]. To help reduce the risk of the emergence and spread of zoonotic pathogens, it would be of great value to encourage the redirection of wildlife hosts to alternate forested lands when they have lost habitat space in a deforested region [44]. The regeneration and reparation of forested lands can reduce the transmission potential of zoonoses while also benefiting the ecosystem as a whole [44]. New studies have shown that viruses are more likely to be transmitted from animals to humans if they are around disturbed ecosystems, which include deforested lands [45]. These outbreaks can lead to an increase in zoonotic pandemics as well. In 2013, the Ebola outbreak emerged after an eighteen-month-old child became ill while playing near a tree and died shortly thereafter [46]. The World Health Organization reported that the Ebola outbreak may have been caused by deforestation, which caused bats to infest the child’s village [47]. Large portions of the bats’ biological environment were destroyed due to deforestation, forcing them to infiltrate the village [48]. Another example is the coronavirus, SARS-CoV-2, which originated in an animal, most likely a bat, and then spread globally causing a pandemic that killed millions of infected patients [49,50]. The rising increase in deforestation causes ecological landscape disruptions, loss of biodiversity, and displaced wildlife migrations to local communities, all of which are associated with increases in zoonotic transmissions [51].

## 4. Tourism and Zoos

As mankind has become more advanced in traveling technologies, the movement of people in large numbers across greater spans of land each day has vastly increased exposure amongst humans, as well as with animals [52]. Furthermore, the exploration of mankind across new locations also creates opportunities for recreational experiences that increase human contact with animals. Interestingly, when people think of animals in contact with humans, sometimes the animals seen around homes on a daily basis, such as birds, domestic pets, and rodents come to mind. In some cases, however, more exotic animals have also come into contact with humans and have increased zoonotic risks in recent years, such as South African lions that are bred on farms for commercial uses, including tourism [53]. On these farms, interactive tourism activities, including petting of the cubs, as well as other uses of these captive lions, such as slaughtering them for trophy hunting or medicinal purposes, have increased direct contact and zoonotic risks with big game animals previously kept rather separated from human interactions [53]. Lions in zoos also present zoonotic dangers. Even though the public may not be directly interacting with them, zookeepers and volunteers caring for them are known to obtain zoonotic infections. For instance, a zookeeper caring for a lion cub in 2015 contracted dermatophytosis from the animal transferring pathogenic fungi to the caregiver [53]. As the potential for the spread of disease from human activities becomes more acknowledged, it can shine a spotlight on the need for increased large-scale surveillance of potential emerging zoonoses. The benefits have already become apparent in studies, such as the first large-scale surveillance of zoonotic flaviviruses in European zoo mammals across a seventeen-year timespan [54]. In this study, West Nile virus, Usutu virus, and tick-borne encephalitis virus exposure were found in zoo mammals [54]. The benefit of surveillance of zoo animals for zoonotic transmission became apparent in that study when they looked at the time span data and noted that Anti-West Nile Virus antibodies were detected in the sample of zoo animals at least one year prior to the first outbreaks that were reported in humans in that region [54].

One aspect of tourism and zoo life that promotes the risks of zoonoses is the concept of petting zoos [55]. Interestingly, petting zoos and tourist parks create a zoonotic risk factor that is similar to one of the driving factors of deforestation, which is the relocation of animals normally in high canopy locations to suddenly being at ground level, and thus in closer proximity to humans [25,26,56]. These types of attractions have placed humans in direct contact with animals through petting and feeding, including the now popular way of taking close selfies with animals [55,56]. While some zoos or tourist sites, such as Gunung Leuser National Park in Indonesia, try to minimize the risks with strict rule sets, such as minimum distance restrictions, studies have shown a major lack of compliance [56]. The desire of people trying to obtain the best social media posts has encouraged a blatant disregard for the rules, including Instagram posts of visitors feeding animals that are not meant to be fed, having direct contact, or having proximity less than the minimum restrictions [56]. It should be noted that the increased spread of wildlife selfies is not simply restricted to parks or zoos, but has become the norm in tourist trips out in the wild in general as well [57]. In one study by the World Animal Protection Organization, an analysis of approximately 34 billion images posted on Instagram from a sample of 700 million people found tens of thousands of wildlife selfies [57]. This shocking discovery has led to the organization developing ecotourist pledges to abstain from baiting or restraining animals for selfies as well as the creation of Instagram pages dedicated to educating the public on animal wildlife [57]. Additionally, Instagram has even added a pop-up feature that appears to demonstrate warnings of illegal wildlife trade if anyone searches for wildlife selfies [57]. Although mankind’s obsession with social media has created some zoonotic risks and unpleasant behaviors towards animals, it should be noted that these platforms have also benefited conservation efforts and helped promote better public opinions towards wildlife in general when used in an appropriate manner [57]. While many of these initiatives aim to protect the welfare and rights of animals, it should be noted that they also subsequently help reduce zoonotic risks by decreasing public interactions with many animals. As public opinion has become more aware of the issues surrounding animal-based tourist attractions, companies such as TripAdvisor have stepped forward to stop the sales of tickets for circuses or other entertainment attractions that include cruelty to animals and direct exposure of humans to wildlife [57].

## 5. Wildlife Exploitation and Trade

In addition to urbanization, deforestation, and climate change, wildlife exploitation heavily contributes to the spread of zoonotic diseases between human vendors or consumers and the wildlife or products they exchange [58,59]. All over the world, wildlife is traded to make food, goods, and traditional medicine as well as to serve as pets [51,60,61]. In addition to food products, such as meat, milk, and eggs, there is a multitude of uses for which people trade animal body parts including hide, horn, scales, bile, and bone [51,59]. Camels are regularly sold both within and internationally from Africa for meat and milk production or live animal usage; pangolins in China for meat and scales; lions in South Africa for tourism, farming, and taxidermy; civets in Asia for food and as pets; frogs and snakes for bile in medicine; goats for hide [51,53,59,62,63]. Wildlife harvesting and the trade of millions of animals annually, both legal and illegal, are extremely popular in numerous regions, especially in Asia and Africa, as primary parts of their culture and livelihood [51,59,64].

There are various players and stages along the chain to make and trade food and goods, from hunters or harvesters to butchers, skinners, transporters, vendors, and lastly, consumers as well as any passersby [61,65]. Zoonotic transmission can occur in many ways along the process, both when the wildlife is still alive, e.g., during harvest or transportation, and already slaughtered, e.g., during slaughter cleanup, carcass collection, defeathering or dehairing, evisceration, or consumption [61]. In addition to animals sold for their products, zoonotic pathogens can transfer from those intended as pets, e.g., when outsiders visit or farmers live on farms with them; veterinarians treat them; pet store employees transport and care for them [58]. The pathogens that infect animals can spread directly through skin-to-skin touch, contact with bodily fluids, animal scratches and bites, or exposure to existing wounds, as well as indirectly through contaminated air, water, hands, utensils, or food [61]. Not only is animal-to-human transmission a major risk, but cross-species animal-to-animal transmission is also a major factor in the emergence and evolution of zoonotic diseases [58,64]. The risk of zoonotic transmission increases when infected animals are shipped and sold away from their native regions, allowing for pathogens to switch hosts [64,66]. Seasonally communal pastures that house groups of cattle or poultry that are otherwise separated for the rest of the year provide shared space to transmit disease, which can then spread to humans in contact [58,65]. Similarly, cross-species transmission is very likely when animals are transported from the farm to the market or imported across countries in unnatural species groupings in unsanitized densely cramped spaces [59,67]. Crowded marketplaces particularly present perfect hubs for transmission between different species of animals and humans [59]. Countless legal and illegal wildlife markets bear stands with too many animals cramped in cages that are too close to one another and to vendors and customers, providing ample exposure to respiratory secretions, feces and urine, feathers, and skin, either from the animals being sold or from stray cats, dogs, and rats [59,65]. Asia’s wet markets have received ample and harsh criticism for their unsanitary conditions of the captive animals and walking spaces of the buyers and sellers, as well as the concurrence of live animals and meat sold simultaneously [68]. Many of the animals carrying the greatest numbers of zoonotic diseases are sold in large quantities with the widest ranges of species and densest human population, increasing the likelihood of transmission between species [59].

Vast research highlights everyday infection risks and transmissions, in addition to distinct zoonotic outbreaks that have impacted the world. A study assessing hundreds of thousands of wild animals imported in the United States determined rabies virus, *Bacillus anthracis*, *Mycobacterium tuberculosis* complex, *Echinococcus* spp., and *Leptospira* spp. as the most widespread agents of risk zoonoses [67]. Because Southeast Asia is considered a hub for emerging zoonoses, a similar study was performed in Indonesia on several thousands of mammalian orders spanning bats, primates, and rodents, in which more than half of the species were reported as hosts for 17 zoonotic viruses [59]. Additional research indicates 44 different pathogen taxa in birds, 47 in mammals, 16 in reptiles, 2 in amphibians, 2 in fish, and 1 in invertebrates as zoonotic hosts [64]. The great number of genera capable of hosting an even greater number of widespread risk zoonoses demonstrates the countless opportunities for zoonotic pathogens to be imported and spread from the wildlife trade [59,67]. Even by excluding unidentifiable pathogens and illegal trades, studies confirm that hundreds of animals can host and spread myriad zoonoses [59,64,67]. Despite the telltale results of the studies in the US and Indonesia, they underestimated the risk of zoonotic disease infection because they focused only on live mammals instead of including other classes (pathogenic diseases can cross between classes or even between higher than the class level) and instead of including dead animals [59,67]. SARS-CoV, Influenza, Hepatitis E, and Issyk-Kul easily infect humans and animals in crowded wildlife markets [59]. Cases of everyday transmission include rabies, often from pets such as dogs and cats, as the cause for 95% of human deaths in Africa and Asia; Middle East respiratory syndrome coronavirus from contact with the bodily fluids of camels that are commonly used in medicine; HIV and Ebola, often linked to the consumption of undercooked meat where many pathogens can survive; Nipah from interaction with domestic animals and consumption of food contaminated by the bodily fluids of infected animals in Malaysia, Bangladesh, and India; Bovine tuberculosis from handling or drinking unpasteurized milk of cattle in the US, Europe, and Asia [51,58,59,69]. Thousands of lions in South Africa have been tested to carry 63 known pathogens that cause 83 potentially zoonotic diseases, e.g., dermatophytosis [53]. *Sparganum* is a common pathogen among snakes and frogs that is transmitted through their bile used in medicine, and *Staphylococcus aureus* is a frequent pathogen hidden in healthy asymptomatic Tunisian camels that is transmitted through consumption of their meat [51,62]. Smuggled parakeets spread chlamydiosis to Belgian officers; African rodents exposed to prairie dogs and then to humans lead to monkeypox virus; illegally imported raw fish transmitted opisthorchiasis to a family; illegally purchased pet turtles passed *Salmonella oranienburg* to their owners in 14 US states [64,70,71,72]. Zoonotic diseases that likely originated specifically from wildlife exploitation are scattered throughout the world’s history of major pandemic outbreaks, especially over the past few decades, particularly Ebola, HIV, avian influenzas (AIV), SARS, and MERS coronaviruses [67,73,74,75]. Solely in the US, the Ebola virus spread from primates imported from the Philippines; monkeypox from rodents from Africa; and HIV suspected from chimpanzees from central Africa [59,67,68]. In several countries in Africa and Asia, AIVs H5N1 and H9N2, which have caused many human deaths, continue to evolve to increase bird-to-human transmission in poultry farming and live bird markets, thereby amplifying the risk of another pandemic [51,65,76]. In 2002, SARS-CoV originated in civets, which became popular household pets and slaughtered openly in China’s wildlife markets [75]. Undeniably, COVID-19 is the most profound and recent zoonotic pandemic outbreak, widely believed to have originated from bats or potentially other animals sold in a live animal market in China––with the first patients to test positive linked to Wuhan’s wet markets––which has shifted the focus of zoonosis studies to wildlife trade [63,64,68].

The recurrence of outbreaks that originated as zoonoses, particularly COVID-19 as the most recent and severe one, has led to a heavy emphasis on the importance of screening and minimizing wildlife trade in an attempt to suppress zoonotic pandemics [60]. However, this goal is extremely complex and challenging to achieve. The nature of the market and the pathogens it carries make the diseases greater in number, diversity, resistance, and infection [64]. Wildlife trafficking greatly decreases biodiversity but leads to the evolution of infectious diseases, consequently increasing zoonotic spillover [51,64,77]. For example, as AIVs persevere and continue to evolve throughout Africa and Asia amid inadequate vaccine distribution due to a lack of financial and human resources, they diversify further, making it harder to record, target, and eliminate [65,76]. Analysis of animal importation and maintenance reports that only select animals are quarantined upon arrival and only a few diseases are tested [67]. Furthermore, some diseases do not cause clinical signs in their infected hosts until later or at all, leading farmers to unknowingly trade early infected animals [58,62]. Few wildlife markets adhere to the code of hygiene, e.g., disinfecting, ventilating, and ensuring accessible clean water, as well as the type of species and the number of animals allowed to be sold [65]. Wet markets have been criticized, but shutting them down will only increase private or illegal trade, which operates in far worse sanitary and ventilation conditions under little to no biosecurity and recording [65,76]. Altering legal trade with illegal will also increase the number of species and individuals sold [59]. Even if proper legislation minimizing wildlife trade were simple, it poses an overwhelming ethical and financial challenge because trade plays a key part in culture and livelihoods [59,68].

After Dr. Fauci called COVID-19 a “direct result” of wet markets, scrutiny of the wildlife trade emphasized that tighter biosecurity and more controlled regulations are needed than they were before the pandemic [60,63]. The confirmation of countless mammals as hosts for zoonotic diseases in several studies has already highlighted the need for stricter surveillance and more sanitary conditions [59,67]. In the past, the Center for Disease Control and Prevention (CDC) restricted the importation of Gambian pouched rats during the monkeypox pandemic, and the Chinese government ordered the mass killing of tens of thousands of infected civets following the SARS outbreak [67,68]. PREDICT, a project of USAID’s Emerging Pandemic Threats program, discovered more than a thousand new viruses in mammals mostly from Asia, Africa, and a few from Latin America [64]. Countries with slaughterhouse inspection in place detects about two-thirds of bovine tuberculosis outbreaks and cities with firm hygiene and animal storage rules, such as Hong Kong, show lower transmission rates of zoonotic pathogens [58,65]. In 2017, the CDC organized the first “One Health” workshop with the US Departments of Agriculture and Interior to address zoonoses, prioritizing influenza, salmonellosis, West Nile Virus, plague, emerging coronaviruses (SARS and MERS), rabies, brucellosis, and Lyme diseases [51]. In an attempt to control human exposure to zoonotic pathogens while maintaining steady wildlife trade, several protocols have been suggested and instituted by the World Health Organization, World Organization for Animal Health, and United Nations Environment Programme, including sanitation, disinfection, prohibition of overnight poultry storage, use of protective equipment, and tracing [59,65]. Ensuing the COVID-19 outbreak, the Chinese and Vietnamese governments prohibited the trade and consumption of wild animals [78,79]. Infectious disease researchers argue that the risks of wildlife trade are becoming increasingly abundant and severe, and the better way to diminish them than regulating the trade is to dissolve it [60]. Patrolling trade is difficult because of poor records, mislabeled species, and the heavy influence of illegal operations on legal ones [60]. Terminating illegal wildlife trade is an issue that so-called developing countries—often the source of illegal bushmeat for food and exotic live animals for pets—and developed countries, such as those in the EU that import animals, alike need to tackle [77].

However, banning the trade will strip thousands of their livelihoods or drive it underground, so strict regulations while maintaining steady trade need to be executed and research aiming to prevent future outbreaks must address deeper-rooted issues of poverty and health [64]. A more effective and favored strategy than illegalizing trade would be to prioritize surveillance, restriction, and sanitary and quantitative control of pathogen and animal taxa known to be prevalent and most infectious in the largest markets first and foremost [59,60,64,67]. Analyzing the dynamics and processes of trading networks—including the animals’ handlers at each step, countries of origin, methods of breeding (wild-caught and captive-bred), grouping during maintenance, transportation, and trade (how many and with which species)—helps ensure protocol observance, better understand and hypothesize zoonotic transmission, and guide new regulations to aid in disease eradication [51,61,65,67]. Additional control includes clear labeling of meat as domestic so as not to disguise any as exotic, which is often illegal [63]; separation between animals of different species and origin [65]; criminalization of trade that does not adhere to environmental rules [77]; tighter border control in the EU, which has reported hundreds of tons of bushmeat on Air France alone from Central and West Africa every year (likely an underestimate because of lax inspection) and Asian ports, which currently have free-trade zones [63,77]. Continuous detection of known pathogens is critical, e.g., *Staphylococcus aureus* in camels in Africa and bovine tuberculosis in birds and cattle [58,62,69]. Public health organizations, such as USAID’s Emerging Pandemic Threats program, have invested billions into disease tracking, analysis, and appropriate response [61,80]. Forensic laboratories and genomic technologies for animal body parts have been growing in the EU to identify illicit wildlife trade [77]. The One Health Initiative should educate farmers, vendors, importers, veterinarians, pet store employees, and animal consumers on the risks of zoonotic diseases and the application of safety and hygiene measures [64,67,68,76]. Under the One Health Initiative, which was formed in 2008 to align human, animal, and planetary health, experts in wildlife, ecology, public health, epidemiology, and sociology should collaborate on research and legislative measures to minimize zoonotic transmission and educate farmers, vendors, importers, veterinarians, pet store employees, and animal consumers on the risks of these diseases and the application of safety and hygiene measures [51,64,67,68,76]. More needed than ever for the welfare of humans and animals alike is thorough screening and legislation, which can be implemented while maintaining steady wildlife trade and exploitation, to not only combat already-existing zoonotic diseases but also to prevent future outbreaks.

## 6. Climate Change

**Figure 2:**
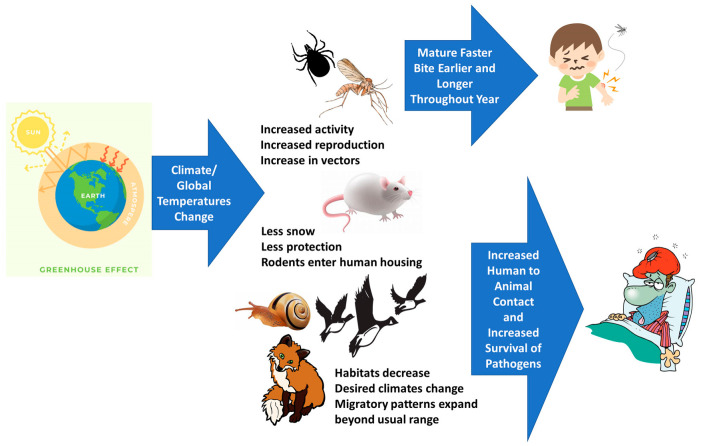
Climate change is more than just temperature change. As temperature, precipitation, humidity, and climate patterns become more disrupted, vector and animal patterns become modified in ways that increase human exposure to infectious diseases.

Finally, it is of interest to briefly mention the connection between zoonotic infections, vector-borne transmissions, and climate change. Although climate change is more of an environmental factor than the other factors discussed in this review, climate change has become a topic of growing concern as more evidence is revealed of the large increases in greenhouse gases due to human activity [81]. These increases have led to abnormal changes in the climate, which has, in turn, affected the occurrence of zoonotic diseases [81]. Changes in global temperatures can alter the number of vectors, transmission cycles, and contact between species, which affects the emergence of zoonotic diseases [81]. If vectors mature faster and last longer throughout the year, for instance, or habitats lose their favorability as precipitation or protection diminishes, then human exposure levels expand consequentially (Figure 2).

The impact of climate change on zoonotic and vector-borne transmissions is most obviously seen with arthropod vectors, which are sensitive to changes in temperature [82]. Mosquitoes show a rise in activity and reproduction when temperatures increase, leading to an increase in vectors [82]. Additionally, the pathogens using mosquitoes as hosts reach maturity faster. In Italy, there was an association between an outbreak of the chikungunya virus, which is spread by mosquitoes, and a higher temperature in the area due to climate change [82,83]. The same effects are seen for ticks and sandflies, and ticks will begin biting earlier than usual in the year and for a larger gap in time [82]. For example, Crimean-Congo hemorrhagic fever (CCHF) is caused by a virus transmitted by ticks, and there was an outbreak of CCHF in Turkey that was correlated with a warmer spring in the country a year earlier [84]. The same observation was made for the *Ixodes ricinus* tick in Sweden, which is a vector for Lyme borreliosis and tick-borne encephalitis (TBE) [85]. Sweden’s climate has become warmer since the 1980s, which has caused the population density of ticks to rise and extend northward [85]. This has been related to the growing incidence of TBE in Sweden [85]. The spread of disease by way of rodents will also increase as temperature increases, causing there to be less snowfall, which rodents use for protection [82]. This will cause rodents to enter human housing, leading to higher chances of transmission between humans and rodents [82,86]. For similar reasons, the risk of rabies in Alaska would increase [87]. This is because foxes usually spread rabies to dogs, which then infect humans, and a decline in sea ice because of climate change could lead to more interactions between foxes and dogs, as foxes lose parts of their habitat [87]. Cryptosporidiosis and giardiasis could also become issues in Alaska, as they are usually transmitted via ingestion of water contaminated with animal feces and human feces, respectively [87]. The change in climate could cause animals with the disease to move into areas north where humans have not been exposed to yet, and it increases rain and the moisture of the soil, which could heighten the survival chance of pathogens [87]. This effect was also seen in China as *Oncomelania hupensis*, a type of snail that acts as a vector for Schistosomiasis, has spread out over its usual range into Northern China due to increasing temperatures [88]. Migratory birds will also shift north due to climate change, which may change the transmission pattern of the highly pathogenic avian influenza H5N1 [89]. Rising temperatures also pose a threat in that they might prompt mutations in pathogens that could increase their reproduction or survival rates [88]. In addition, with an increase in temperature, climate change can lead to very dry conditions in certain parts of the world, such as Arizona, U.S.A [90]. As the climate there has changed, there has been an association between the dry weather and the incidence of coccidiomycosis [90]. As observations have shown, the alterations in the number and population of species present in communities can lead to an increase in the spread of zoonotic disease-causing pathogens [91]. However, it could also lead to a decrease, as additional species in a community may not be viable host options, causing a decrease in the spread of pathogens [91].

One type of pathogen in particular that can have increased transmission due to changes in climate is foodborne pathogens [92]. This is because the optimal temperature for the majority of foodborne pathogens is around 37 °C, so as temperatures warm, these pathogens’ growth rate increases [93]. Additionally, some pathogens, such as *Listeria monocytogenes*, are inhibited by freezing events and climate change will lessen the frequency of these events [93]. As a result, there will be an increase in pathogen populations in habitats that did not have issues before because of inhibitory freezing events [93]. Moreover, in the European Union, there was a positive correlation between the possibility of contracting campylobacteriosis and the mean weekly temperature [92]. There has also been a correlation between increases in salmonellosis notifications and weekly temperature increases for temperatures above 5 °C [92].

Furthermore, the increasing temperatures due to climate change have not only affected outbreaks directly but also indirectly by causing an increase in heavy rainfall [92]. This has been correlated with breakouts of waterborne norovirus, as increasing precipitation causes wastewater overflow to taint aquatic habitats [92]. In addition, the impact of heavy rainfall was observed after the 2015–2016 El Nino-Southern Oscillation (ENSO) event [94]. It has caused major rises in precipitation in different parts of the world and led to higher incidences of plague in Colorado and cholera in Tanzania due to that [94]. The rise in cholera could be because an increase in flooding would lead to more water contamination and this added stress on aquatic environments may make them more vulnerable to infection [94]. Climate change has also caused early winters in Northern Europe to become rainier, which is associated with a rise in the transmission of Puumala orthohantavirus (PUUV) in bank voles [95]. This could lead to a rise in the risk of transmission to humans [95]. The exact mechanism for this is not known, but some possibilities are that the change in winter has modified host behavior or physiology, or increased the period of time the pathogens persist in the environment [95]. An example of this is the outbreak of nephropathia epidemica, which was caused by PUUV in Sweden in 2007 [95]. In the autumn before the outbreak, there was a higher number of voles, and then a very rainy winter where many voles began infesting houses, likely leading to the jump in cases of nephropathia epidemica [95]. Also, in Kenya, there was an increase in *Aedes aegypti* mosquito eggs and adults during months with higher-than-normal rain [96]. These mosquitoes are vectors for dengue fever, so the increase in rain could present a concern for a potentially higher risk of the disease [96]. Another facet to the issue of global warming is not only that it will push species further inland to human habitats, but that it will also expose pathogens that were frozen [83,97]. In Yakutia, Russia there have been outbreaks of anthrax in areas near burial sites that are thought to be caused by the pathogen surviving in permafrost soils [97].

The importance of climate change’s impact on the emergence of zoonotic diseases has multiplied in recent years as the world has become more aware of the dangers of increased zoonotic transmissions through experiences such as the COVID-19 pandemic [98,99]. It has been suggested that COVID-19 was spread from a seafood market in Wuhan, China, and it has been recorded that wild animals can be hosts for SARS-CoV-related coronaviruses [98,100]. While no direct links have yet been made between climate change and COVID-19 emergence, it is an area that is currently seeing hypotheses developed. Ultimately, it is believed that climate change will lead to further intrusion into existing habitats for animals and more interactions with humans, which can lead to more zoonotic infections [98].

## 7. Conclusions

As mentioned above, it has been estimated that 61% of the microbial infections already known to plague humans are of zoonotic origins, and that up to approximately 75% of emerging infectious diseases worldwide are zoonotic as well [101]. These diseases have put a major strain on healthcare systems, financially burdened global economies, and cost numerous lives [101]. As we have demonstrated in this review, these zoonotic diseases have become more and more common due to various factors, such as urbanization, deforestation, tourism, zoos, climate change, and wildlife exploitation, changing the way that animals and humans live their daily lives and interact with each other.

Urbanization, tourism, climate change, and deforestation have contributed to an increase in human–animal contact as habitats are lost, causing animals to venture further into human housing and heightening transmission risks [7,18,45,51,82,86]. Wildlife trade has also led to a rise in contact, which can occur during several different parts of the trade from transportation to consumption [61]. All of these factors have similarities in how they further zoonotic disease emergence, but also present unique issues (Table 1). One of the recurring themes, for instance, in the human-related factors that drive zoonoses is that animals that were far outside of the reach of humans in locations such as canopy levels of forests have now been forced towards ground-level interactions with humans [30,53,55]. Furthermore, ecological landscape changes have modified the temperature and moisture contents in the environment, as seen with climate change, but also with deforestation, creating sunlit pools in demolished lands [25,26,82]. These changes, in turn, promote an increase in vectors that transfer pathogens, while also driving new migrations of animals towards human dwellings, as protection levels or food sources vary with the new landscapes [25,26,82]. It is important to continue to study these patterns and elucidate the intricacies of human–animal interactions and transmissions to better develop policies, urban risk reduction management strategies, including pest management, public health awareness, environmental hygiene campaigns, and preventive measures to reduce future outbreaks. This value has already been seen, for instance, when a study by the World Animal Protection Organization shined the light on the growing problem of wildlife selfies being taken for social media. Once this potential factor of zoonotic transmission was highlighted, companies such as Instagram and TripAdvisor were able to step forward and increase practices to reduce risky wildlife behaviors and also promote public awareness and education [57].

Hopefully, more organizations will continue to spotlight the growing actions of humans that are risking zoonotic transmissions and help reduce the spread of epidemics in the future. New online tools have already been developed, such as for preventing Ebola virus spillovers in Africa, compiling datasets, and building niche maps to track potential reservoirs of zoonotic diseases [102]. Norway has also taken steps to reduce zoonotic risks after studying the human factors impacting zoonoses. For instance, they determined that burrowable, unpaved regions are critical for Norway rat health and presence in urban regions so they began pavement, drainage, and landscaping plans, especially in streets and vacant lots or yards, to minimize the environmental carrying capacity and reduce rat infestation potentials [103]. Furthermore, as more studies and reviews explore the connections between human health, wildlife health, and ecosystem health, we will gain a better and broader perspective on public health threats and mitigation techniques that go beyond just a narrow or linear focus [104]. So often people focus on the linear concept of humans becoming infected by animals carrying pathogens that it is often neglected that there actually exists a bi-directional transmission possibility that increases the risks of infections to humans and animals [105]. The bi-directionality of transmission and the existence of reverse zoonosis were rarely seen until COVID-19 shined a spotlight and demonstrated the need for more rigorous and expansive microbial testing to be considered [105]. Overall, the connections between animal health, human health, and ecosystem health need to be more carefully explored. Advancements such as microbiome surveillance and analyses can become valuable tools in these explorations, as environmental and host-associated microbiomes have potential as early warning indicators of ecosystem and wildlife health and are known to respond to anthropogenic disturbances and management interventions [106]. There is no singular answer as to how to prevent and minimize zoonotic infection emergences and transmissions, but hopefully, through many collaborative answers, we can create a more mindful and safer future.

## Figures and Tables

**Table 1:** The driving forces of zoonotic disease emergence and their main impacts.

Driving Forces	Main Impacts	References
Urbanization	▪ Modification and loss of habitats lead to animals infiltrating further into urban areas ▪ Lowers biodiversity which can allow vectors to dominate areas ▪ Higher risk of parasitic contamination or transmission	[7,8,10,14,15,18,21]
Tourism and Zoos	▪ Exotic animals previously not in contact with humans are now directly exposed to or interacting with humans ▪ Animals normally in high canopy locations suddenly at ground level near humans ▪ Petting zoos and social media promote petting, touching, feeding, and taking selfies with animals	[25,26,53,55,56]
Climate Change	▪ Increased number of vectors, and vector reproduction and activity ▪ Loss of habitats and preferred climates lead to more vectors entering human housing ▪ Switching of hosts due to changes in migratory patterns	[82,83,89,90,95]
Deforestation	▪ Loss of biodiversity allows for pathogens to overshadow areas ▪ Ecological landscape disruption removes habitat layers of forest and encourages animal migration towards urban developments ▪ Loss of canopy layer of the forest removes protection from sun and rainfall, causing soil erosion ponds that breed vectors ▪ Higher potential for zoonotic spillover	[33,44,45,47,48,51]
Wildlife Exploitation and Trade	▪ Increased human–animal contact ▪ Pathogens can switch hosts during transportation ▪ Risk of cross-species animal–animal transmission	[58,61,64,66]

## Data Availability

Not applicable.
